# Characteristics analysis of *Early Responsive to Dehydration* genes in Arabidopsis thaliana (*AtERD*)

**DOI:** 10.1080/15592324.2022.2105021

**Published:** 2022-08-02

**Authors:** Guofan Wu, Nongfu Tian, Fawen She, Aohua Cao, Wangze Wu, Sheng Zheng, Ning Yang

**Affiliations:** Laboratory of the Research for Molecular Mechanism and Functional Genes of Plant Stress Adaptation, College of Life Sciences, Northwest Normal University, Lanzhou, China

**Keywords:** *Arabidopsis thaliana*, *AtERD* genes, bioinformatics, signal pathway

## Abstract

Early Responsive to Dehydration (ERD) genes are rapidly induced in response to various biotic and abiotic stresses, such as bacteria, drought, light, temperature and high salt in *Arabidopsis thaliana*. Sixteen ERD of *Arabidopsis thaliana* (*AtERD*) genes have been previously identified. The lengths of the coding region of the genes are 504–2838 bp. They encode 137–745 amino acids. In this study, the *AtERD* genes structure and promoter are analyzed through bioinformatics, and a overall function is summarized and a systematic signal pathway involving *AtERD* genes is mapped. AtERD9, AtERD11 and AtERD13 have the GST domain. AtERD10 and AtERD14 have the Dehyd domain. The promoters regions contain 32 light responsive elements, 23 ABA responsive elements, 5 drought responsive elements, 5 meristem expression related elements and 132 core promoter elements. The study provides a theoretical guidance for subsequent studies of *AtERD* genes.

## 1 Introduction

Plants can’t avoid stresses as effectively as animals due to their inherent growth characteristics. However, the plants develop some adaptive mechanisms to minimize the damage caused by the stresses, from stress perception to stress response.^1^

Among all kinds of stresses, the proportion caused by water shortage is the highest^2,3^ and the water shortage caused by drought is most serious. To overcome drought stress, plants have evolved three major adaptive mechanisms including drought escape, drought avoidance and drought tolerance.^4^ A large scale of strategies are to prevent water loss under drought conditions, and thus to balance the optimal water supply of important organs for plants resisting drought.^5,6^ Firstly, the root responds to the change of soil water content on the scale of cells and the whole root systems. The niche of root stem cells, meristems and vascular systems are coordinated under drought stress.^7,8^ Secondly, at the cellular level, drought signal would promote the production of proline, trehalose and other metabolites, which would, inturn, stimulate the antioxidant system to maintain redox balance and prevent cell damage and membrane integrity damage caused by oxidase.^7^ In addition, drought signal also stimulates the response of plant hormone pathways, such as abscisic acid (ABA), salicylic acid (SA) and JA. All these strategies are complex and controlled by multiple genes and ways.^9–11^ The *Early Responsive to Dehydration* (*ERD*) genes are defined due to plants fast respond to drought stress^12^ and activated in the early stage of drought stress.^13^

To date, a total of 16 *ERD* genes of *Arabidopsis thaliana* (*AtERD*) named *AtERD1-AtERD16* are annotated. They come from different subfamilies with various functions^14^ including chloroplast ATP protein dependent enzyme, heat shock proteins (HSP), proline dehydrogenase, glycocarrier protein, Glutathione S-transferase family proteins, allene oxide cyclase, hydrophilic protein lacking cysteine, ubiquitin (UBQ) protein.^15^ However, less attention has been paid to a comprehensively analyzing about *AtERD* genes overall function in previous studies. This study summarizes and analyzes the overall function ranging from genes structures, promoter sequences to the whole signal pathways.

## 2 Gene structures and promoters analysis of *AtERD* genes

### 2.1 Gene structures of *AtERD* genes

**Figure 1.**
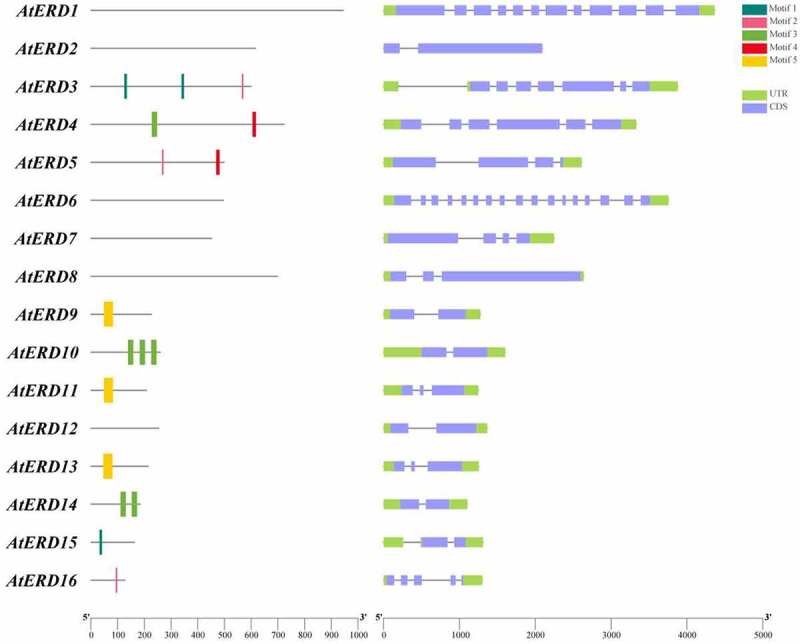
Conserved domain (left) and structure analysis (right) of 16 *AtERD* genes. The conserved domain and structure analysis are drawn with TBtools. Motif 3 is Dehyd domain and motif 5 is GST domain. Motif 1, motif 2 and motif 4 do not have consistent domains in the actual analysis.

The conserved domains and gene structures of *AtERD* genes are analyze by TBtools according to the information from *Arabidopsis thaliana* Tair database (https://www.arabidopsis.org/) and SMART (https://smart.embl.de/) (Figure 1). It has been found that AtERD9, AtERD11 and AtERD13 have the GST domain; AtERD10 and AtERD14 have the Dehyd domain; AtERD3 have Methyltransf_11 domain, and AtERD4 have RSN1_TM5, PHM7_cytand RSN1_7TM domain. AtERD5 have Pro_dh domain, and AtERD16 have a UBQ1 domain. Relatively, in the subgroup from *AtERD1* to *AtERD8*, longer sequence and more exons are contained in each gene, which makes it possible for them to edit more functional proteins, especially in *AtERD1, AtERD3* and *AtERD6* (Figure 1).

### 2.2 Promoters analysis of *AtERD* genes

**Figure 2.**
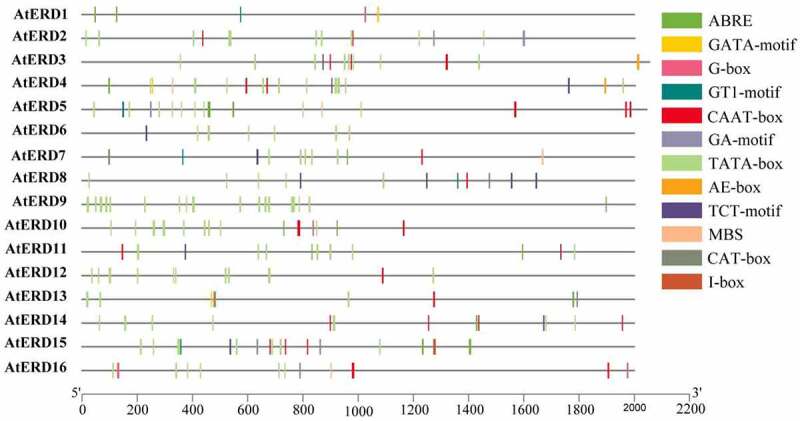
Cis-elements in promoter regions of 16 *AtERD* genes. The elements are analyzed from 2000 bp upstream promoter regions and draw them with TBtools. ABA responsive element: ABRE; Light responsive element: GATA-motif, G-box, GA-motif, GT1-motif, AE-box, TCT-motif, I-box; Drought responsive element: MBS; Common cis-acting element: CAAT-box; Core promoter element: TATA-box. Meristem expression related element: CAT-box.

In order to get insight into the response factors of *AtERD* genes, the promoter sequences from the Tair database (https://www.arabidopsis.org/) are analyzed by PlantCARE (http://bioinformatics.psb.ugent.be/webtools/plantcare/html) (Figure 2). It is showed that the *AtERD* genes generally respond to ABA, light and drought. There are 32 light responsive elements except *AtERD9* and *AtERD12*, 23 ABA responsive elements except *AtERD2, AtERD3, AtERD6, AtERD8* and *AtERD12*, 5 drought responsive elements in *AtERD4, AtERD5, AtERD7* and *AtERD16* and 5 meristem expression related elements in *AtERD7, AtERD8, AtERD13, AtERD15* and *AtERD16*. The core promoter element TATA-box is found in all promoters, and a total of 132 TATA-boxes are distributed in different promoters. However, conclusion cannot be drawn that *ERD* gene without response element means it cannot play a role in ABA, light or drought. It maybe responds to the corresponding stresses through the indirect process of other unknown pathways.

## 3 Associated functions of *AtERD* genes

**Figure 3.**
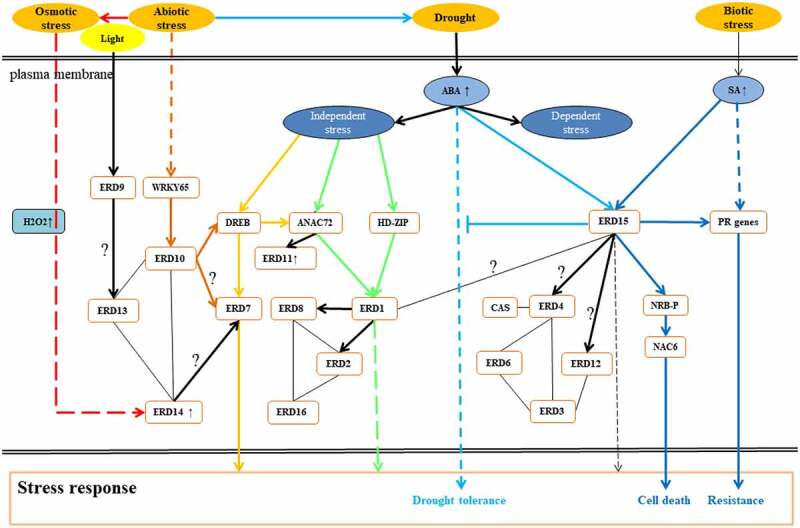
The expression pathway pattern of *AtERD* genes. The Lines represent interactions between proteins, and the arrows represent downstream regulation. The solid line represents direct action and the dotted line represents indirect action. The question mark indicates that it is not clear whether it has a direct effect on the way. Abbreviations used for genes: DREB, dehydration-responsive element binding protein; RD29A, responsive to desiccation 29A; HD-ZIP, homeodomain leucine zipper; ANAC72, Arabidopsis NAC domain containing protein 72; PR genes, suppressor of auxin resistance 1; CAS, calcium sensing receptor; WRKY65, WRKY DNA-binding protein 65.

The overall information of *AtERD* genes is summarized and shown in Table 1. The subcellular localizations of the *AtERD* genes show that the majority localizations are in the nuclear, cytoplasm and membrane. Their main functions include transcription factors (TFs), HSP, GST and others. Based on the functions and existing research, signal pathways revealing the functions of *AtERD* genes are illustrated by Scienceslide (Figure 3). The detailed functions of *AtERD* genes are as follows.

**Table 1.** Characterization of 16 *AtERD* genes.

Gene	Gene ID	Other name	CDS/bp	Theoretical (pI)	Molecular weight (KD)	Location	References
*AtERD*1	AT5G51070	K3K7_27; SAG15	2838	5.89	103.23492	nuclear; chloroplast	^16^
*AtERD*2	AT1G56410	F13N6_9; HSP70T-1	2580	5.22	68.35671	cytoplasm	^17^
*AtERD*3	AT4G19120	T18B16_90	2769	7.44	68.33094	cytoplasm	^18,19^
*AtERD*4	AT1G30360	T4K22_4	2613	9.28	81.93513	plasma chloroplast, vacuole	^20,21^
*AtERD*5	AT3G30775	PDH1; PRO1; PRODH;	2751	6.41	54.9558	inner mitochondrial membrane	^22,23^
*AtERD*6	AT1G08930	F7G19_19	2742	8.87	54.35499	cytoplasm	^24,25^
*AtERD*7	AT2G17840	T13L16_14	2742	5.30	49.00035	LDs and the cytosol	^26,27^
*AtERD*8	AT5G56030	HSP81.2; HSP90.2; MDA7.7;	2727	4.95	80.06406	cytoplasm	^28–30^
*AtERD*9	AT1G10370	GST30; GST30B; GSTU17	2589	6.20	25.3073	cytoplasm	^31,32^
*AtERD*10	AT1G20450	F5M15_21; LTI29; LTI45	1887	5.11	29.54786	cytoplasm	^33–35^
*AtERD*11	AT1G02930	ATGST1; ATGSTF3; ATGSTF6; F22D16_7; GST1	2037	5.80	23.48593	Cytoplasm	^36^
*AtERD*12	AT3G25760		2277	9.11	27.80154	Cytoplasm	^37^
*AtERD*13	AT2G30870	ATGSTF10; ATGSTF4; F7F1_8;	1437	5.49	24.23	Cytoplasm	^36^
*AtERD*14	AT1G76180	T23E18_12	600	5.40	20.78638	Cytoplasm	^38–40^
*AtERD*15	AT2G41430	CID1; LSR1; T26J13.2	1032	4.51	18.41114	nuclear	^12,41^
*AtERD*16	AT3G52590	F3C22.8; HAP4; HAPLESS 4;	504	9.94	14.73339	cytoplasm	^22^

### 3.1 Transcription factors: *AtERD1, AtERD15*

AtERD1^23,42^ and AtERD15^12,43^ are located in the nucleus as transcription factors, AtERD1 contains a putative chloroplast-targeting signal at the N-terminus.^16,44^ Studies have shown that the expression of *AtERD1* gene is not only induced by dehydration and high salt,^16^ it is also influenced by natural aging, dark-induced differentiation and chlorosis.^42^ When induced by drought, the expression of *AtERD1* is independent of ABA pathway^42^ (Figure 3, green lines). *AtERD1* is also identified as a typical stress response marker gene under drought stress. Other genes that act as the marker same as *AtERD1* include *ABI1*, *DREB*, *KIN2*, *RAB18*, *RD20*, *RD29A* (*Responsive to desiccation 29A*) and *RD29B*.^45^ Among them, AtERD1 and RD29A are not affected by ARR8,^46^ however, they indirectly respond to stress through other genes like NAC72^47^ to activate downstream drought resistance genes.^47^

*AtERD15* is a member of dehydration-stress-induced genes in *Arabidopsis thaliana*. It encodes a small acidic protein rapidly responding various biotic and abiotic stresses,^13,31^ such as dehydration, salt and low temperature, external damage, ABA, SA, plant pathogens.

In many studies, it has been found that *AtERD15* is negatively regulated by ABA signal. It can prevent the rapid response of plants to biological stress, and can be used as a buffer to weaken ABA response, so as to reduce the damage to plants. When plants feel different signal stresses, they will cause the changes of hormones. These changes lead to the corresponding change of *AtERD15* gene expression, which inducing in turn the expression of some downstream genes, and finally improving the stress resistance of plants. ABA pathway (Figure 3, blue lines) and SA mediated injury defense pathway are two opposite pathways. However, *AtERD15* is responded to both ABA and SA, which means that *AtERD*15 may be a transfer station regulated by multiple signals including H_2_O_2_ signal.^48,49^

Biological stresses on plants are mainly caused by various pests and pathogens, such as bacillus subtilis, fungi and oomycetes, bacteria and phytoplasmas and viruses.^50^ It is usually caused by infection and competition. When plants suffer from biological stresses, some anti-bacteria substances will be induced. The secretion of these substances requires continuous messenger and gene response. *AtERD15* is found to be involved in the process of biological stress^31,48^ (Figure 3, deep blue lines). *AtERD*15 may also be involved in the weakening of stomatal response to ABA controlled by the core ABA signal module. It has not reported that whether other *AtERD* genes have relevant regulation in biological stresses.

Studies on *ERD15* genes of other plants found that the *SpERD15* of *Pansanum Penellii* enhances the accumulations of soluble sugar and proline in transgenic plants mainly through enhancing osmotic regulation, and coordinates the expression of stress-related genes to improve the drought resistance.^51^ Overexpression of *VaERD15* gene can improve the cold resistance of transgenic plants.^52^ Moreover, its interaction genes *XTH7, GS, RPS23* and *LQY1* jointly regulate the response of black grape to low temperature stress. When *VaERD15*^52^ and *ZmERD4*^53^ are transformed into *Arabidopsis thaliana*, the resulting transgenic lines show enhanced tolerance to freezing injury, drought and salt stresses. In addition, *MsERD15* gene induced by ABA in alfalfa can respond to the induction of SA, and participate only in the initial response of plant defense caused by MeJA. It is also speculated that *MsERD15* gene may participate in the formation of autumn dormancy of alfalfa through the light response process and ABA signal process [Fu ^54^]. Meanwhile, *GmERD15* plays a role in cell death. It is the upstream component of NRP mediated signal induced by endoplasmic reticulum stress, which connects endoplasmic reticulum stress with cell death signal induced by osmotic stress.^55^

### 3.2 HSP: *AtERD2, AtERD8*

In fact, plants are dependent on HSPs in adapting to heat stress. HSPs are divided into five subfamilies according to molecular weight, including small HSP (SSP), HSP60, HSP70, HSP90 and HSP110.^56,57^ HSPs play a role not only in maintaining cell balance, but also stabilizing protein folding and preventing from polymerization. With the help of some HSPs such as HSP60, HSP70 and HSP90,^58^ non-native proteins keep in a competent state for subsequent refolding. By the medium of Hsp100/Clp, the aggregates formed by the denatured or misfolded proteins are further resolubilized and followed by refolding or degradation by proteases.^59^ Some HSPs chaperones like Hsp70 and Hsp90 accompanying the signal transduction and activating some specific transcription factors lead to the synthesis of other members of HSPs/chaperones. *AtERD2* and *AtERD8* are proved to encode two heat shock proteins: HSP70T-1 and HSP81.2 (HSP90.1).^60–62^ The expression of *AtERD2* and *AtERD8* can be activated by *AtERD*1. In this subnet, *AtERD16* (Ubiquitin-60S ribosomal protein L40-1) is proved to be involved in as the HSP cognates and its expression is affected by dehydration stress instead of ABA^31^ (Figure 3, ABA independent stress).

### 3.3 GST: *AtERD9*, *AtERD11 and AtERD13*

Some *AtERD* genes do not directly respond to hormones. *AtERD9* is involved in light signal that mostly consisting of phyA-mediated photomorphogenesis. It is involved in the integration of ABA signals to modulate various aspects of plant development by affecting glutathione pools.^36,63^ *AtERD11* and *AtERD13*, encoding glutathione S-transferases (GSTs), are not affected by 2,4-dichlorophenoxyacetic acid, 6-benzylaminopurine, ABA, or gibberellic acid (GA).^36^ *AtERD13* and *GST8* differ in their regulations by auxins, cytokinins, jasmonate and low temperature according to the kinetics of their response to wounding. Although several other functions of GSTs have been postulated,^36^ the precise physiological roles remain unknown. After drought and salt treatments, *ERD11* is up-regulated in *NAC72* plants but down-regulated in *OEPeNAC034* and *AtAF1/PeNAC034* plants.^64^

### 3.4 *AtERD10* and *AtERD14*

*AtERD10* and *AtERD*14 have the same dehyd domain and about 70% sequence homology. They are also very similar to ABA induced class II LEA proteins. *AtERD10*, encoding glutathione transferase under stresses,^39^ is located in cytoplast.^65^ ERD10 is a highly hydrophilic^32^ and inherently disordered protein (IDPs), which is expressed in some very active division tissues of plants and is ubiquitous under drought conditions.^39^ It is also a typical representative of IDPs.^66,67^ ERD10, COR47 and RAB18^68,69^ are interact with AtPIP2B, a membrane protein. Furthermore, *AtERD10* and *COR6.6* are *DRE*^70^ regulating genes, and their expressions are induced in response to ethylene and HLS1 transcription level.^71^ It can not only protect plants in cold and dehydration, but also play a role in seed development and germination.^32^

*At*ERD14 and its homologue *At*ERD10 are effective chaperones to protect some enzymes,^72^ such as alcohol dehydrogenase, citrate synthase, lysozyme and firefly luciferase, and prevent the enzymes from loss of activity and aggregation. On the other hand, the accumulating of *At*ERD10 and *At*ERD14 help *Arabidopsis thaliana* in response to high salt, drought and low temperature.^29^ For example, *At*ERD14 can accumulate with the increase of hydrogen peroxide content under osmotic stress (Figure 3, Red line). *At*ERD14 also belongs to class II LEA proteins (dehydrogenase)^73^ with K2s domain.^43,72^ Recombinant HSP90 and *At*ERD14 can interact in E. coli even at low temperature.^29^ In addition, *At*ERD14 has ion binding activity in the phosphorylated state, mainly binding calcium ions^38^ and iron ions. Phosphorylation in *At*ERD14 fragment is involved in the regulation of dehydration subcellular localization in stress response.^40^ Moreover, *At*ERD14 may play a role in redox homeostasis during osmotic stress response.

### 3.5 Other *AtERD* genes

*AtERD3, AtERD4, AtERD5, AtERD6, AtERD7* and *AtERD12* are different from other *AtERD* genes. They have no consistent protein expression and similar homologous structures. So they are listed separately.

*AtERD3* encodes an S-adenosyl-L-methionine-dependent methyltransferases protein. The bioinformatic analysis shows that ZmERD3 protein has one specific hit of methyltransferase and a high probability of location in the cytoplasm.^19^ Furthermore, there are many cis-regulatory elements responsive to light, heat, cold, dehydration, as well as other stresses in *ZmERD3* promoter sequence. However, there is only light responsive element in *AtERD3*.

AtERD4 as a hypertonic gated nonselective cation channel or mechanically sensitive ion channel can convert mechanical stimulation into an ion flow penetrating calcium ions.^53,74^ Moreover, AtERD4 also interacts with CAS.^75^ *ZmERD4* is constitutively expressed in different tissues and could be induced by drought stress and salt stress. It also responds to abscisic acid treatment, but low temperature does not induce *ZmERD4*. In addition, compared with wild-type plants, 35S:*ZmERD4* transgenic plants show stronger water tolerance and high salt tolerance.^53^

In addition to ABA and SA pathways, some *AtERD* genes also have other response processes. *AtERD5* encodes methylenetetrahydrofolate reductase (a proline oxidase) located in the inner mitochondrial membrane and is described as a negative regulator of ABA signal.^22,23^ Proline content is one of the most common osmotic indexes in water stressed plants. Its accumulation in dehydrated plants is caused by the activation of proline biosynthesis and the inactivation of proline degradation.^22,76^ *AtERD5* is localized in the mitochondrial intima and is induced by osmotic stress. The sequence analysis shows that the protein encoded by *AtERD*5 is the same as that of yeast *PU7y* gene and the drosophila sluggish-A gene.^22^ They encode the precursor of proline dehydrogenase and are regulated in the mRNA accumulation level of dehydrated and rehydrated plants.^22^

Plants response of drought also implies the carbon allocation to sink organs and sugar partitioning between different cell compartments, and requires the involvement of sugar transporters (SUTs).^77^ *AtERD*6, encodes a putative sugar transporter, is up-regulated by drought and low temperature^24^ and is repressed in leaves by high salinity and ABA.^78^ So far, more researches have been done on early response to dehydration six-like (ESL) than *AtERD6*. With 19 members in *Arabidopsis thaliana*, the ESLs form the largest subfamily of monosaccharide transporters (MSTs) and a common feature is their involvement in plant response to abiotic stresses, certainly including the water deficit. For example, *AtESL1* (*AtERD six-like 1*) is a low affinity facilitator, which is able to transport different hexoses (glucose, fructose, galactose, mannose, and xylose) across the tonoplast. Its expression is highly up-regulated by high salinity and ABA in roots and slightly induced by drought.^78^

*AtERD7* is expressed in lipid droplets (LDs) and cytosol. *AtERD7* belongs to a six-member family, which is separated into two distinct subfamilies. Lipid droplets existing in all kinds of life are neutral-lipid-containing organelles and coated with proteins that carry out a vast array of functions.^26^ *At*ERD7, locating on the LD surface, may be involved in functional aspects of plant stress response.^26^ It plays a role in membrane lipid remodeling during cold stress response in *Arabidopsis thaliana*. Under the normal growth conditions, although the role of *AtERD7* in stress-induced LD dynamics is not excluded, its expression shows no significant changes in the number or morphology of LD.^27^ *AtERD7* and other stress response genes including *COR47*,^35^ *LEA6*,^79–81^ *RAS1* and two hormone signal transduction related genes (*JAZ7* and *PYL5*) are identified as the possible target genes of *ZAT18*, a nuclear C_2_H_2_ zinc finger protein transcriptionally induced by dehydration stress. *ZAT18* overexpression can improve the drought tolerance of *Arabidopsis thaliana*, and its mutation leads to the reduction of plant tolerance.^82^

*AtERD12* encodes a protein similar to allene oxide cyclase and has been poorly studied.^83^ It is one of four genes that encode this enzyme in *Arabidopsis thaliana* and its expression is induced during senescence that involving JA signaling pathway.

## 4 Conclusion

Early dehydration-induced gene expression activation in plants subjected to sudden drought stress^52,84^ reflects the stress response of plants during sudden dehydration. The sequences of these 16 *AtERD* genes do not have consistent conserved sequences, and they play roles in different pathways (Figure 3). According to their promoter analysis results, the cis-acting elements are relatively more ABA and photoresponsive elements. For example, *AtERD1* and *AtERD15*, coding transcription factors associate with drought, have no drought-related elements (Figure 2). This suggests that *ERD* genes are not first-order messengers of drought response,^48^ and they are induced by other related genes (Figure 3).

As research continues, the study of *ERD* genes had made progress and verified the function of different plant *ERD* genes members, such as *Vitis amurensis*,^52^ *Glycine max*,^85,86^ *Zea mays*,^19^ *Betula platyphylla*.^14^ However, there is still a lack of systematic research and specific regulatory network mechanisms about *ERD* genes. In the whole signal pathway, the accurate role of each gene to the other of a pair is not clear, such as *ERD9* and *ERD13, ERD10* and *ERD7, ERD14* and *ERD7, ERD15* and *ERD4, ERD15* and *ERD12* and other interacting genes (Figure 3). According to previous studies, they are speculated that they may have regulatory role between *AtERD* genes. But they need further exploration whether they are positive or negative regulation and whether they are direct or indirect regulation. Furthermore, more functions of plant *ERD* members will be explored to realize regulatory network and functional verification of *ERD* genes in various physiological processes.
